# A case report and literature review: Diagnosis of pelvic retroperitoneal angiomyofibroblastoma assisted by next-generation sequencing

**DOI:** 10.3389/fonc.2025.1560543

**Published:** 2025-09-10

**Authors:** Yue Meng, Xiaomeng Shi, Qianshi Zhang

**Affiliations:** ^1^ Department of Gastrointestinal Surgery, The Second Affiliated Hospital of Dalian Medical University, Dalian, China; ^2^ Piedmont Urgent Care by Wellstreet, Atlanta, GA, United States

**Keywords:** angiomyofibroblastoma, pelvic neoplasm, differential diagnosis, molecular adjunct, next-generation sequencing

## Abstract

Angiomyofibroblastoma (AMFB) is a rare benign soft tissue tumor that primarily arises in the vulvovaginal region of women of reproductive age. Due to its rarity, AMFB is often overlooked in the differential diagnosis, and as such, it is not frequently encountered in clinical practice. Here, we present the case of a 29-year-old female with a pelvic retroperitoneal tumor, confirmed postoperatively as AMFB based on histopathological and immunohistochemical findings. Additionally, we highlight the utility of next-generation sequencing (NGS) as a valuable tool in the differential diagnosis of such rare tumors. Due to the tumor was located at the narrow part of the pelvis and was adjacent to important tissues and organs such as the uterus and bladder, we performed surgical resection using the Da Vinci robotic system after obtaining the patient’s informed consent. The procedure was successful, with no complications observed during the three-month postoperative follow-up. Furthermore, we reviewed the literature on AMFB, focusing on reported cases to identify and summarize patient characteristics, clinical presentations, histological features, and diagnostic challenges associated with this rare tumor. We aim to offer a reference for clinicians facing similar cases, aiding in accurate diagnosis and management, while addressing the challenges posed by the rarity and diagnostic complexity of angiomyofibroblastic tumors, ultimately improving clinical outcomes and advancing knowledge.

## 1 Introduction

Pelvic tumors, due to their unique location and unclear origin, present a significant health concern, particularly for women. The incidence of primary retroperitoneal tumors is 0.5-1.0 per 100,000 individuals (1). Imaging can help identify the site of the tumor and the distribution of nearby neurovascular structures, which may offer preliminary insights into its origin and nature. However, histopathology remains the gold standard for definitive diagnosis (1–3). Angiomyofibroblastoma, first reported in 1992, is a rare, slow-growing interstitial tumor, predominantly benign (4). Fewer than 150 cases have been reported. It most commonly occurs in the lower reproductive tract of women of reproductive age. In men, it is rarely reported and typically presents as a slow, painless growth (4–9). Accurate diagnosis, careful assessment of surgical options, and the preservation of vital tissues and organ functions remain crucial areas of ongoing discussion and research. This article presents a case of robot-assisted retroperitoneal laparoscopic resection of a pelvic mass, which was postoperatively diagnosed as an AMFB through histopathological analysis and confirmatory genetic testing. By reviewing the existing literature on AMFB, we aim to elucidate its clinical characteristics and propose insights for optimizing diagnostic and therapeutic strategies.

## 2 Materials and methods of NGS

### 2.1 DNA extraction and library preparation

Sample processing and genomic profiling were conducted in a laboratory accredited by the Clinical Laboratory Improvement Amendments (CLIA) and the College of American Pathologists (CAP) at Nanjing Geneseeq Technology Inc., Nanjing, China, as previously described (10, 11). Genomic DNA was extracted and quantified from tumor specimens and control samples using the Qubit 3.0 fluorometer. Library preparation was performed using the KAPA Hyper Prep Kit (KAPA Biosystems, Wilmington, MA). Target enrichment was achieved with customized xGen lockdown probes (Integrated DNA Technologies, Coralville, IA) designed to target 425 genes relevant to cancer and radiotherapy response (Radio-tron gene panel, Nanjing Geneseeq Technology Inc.). Hybridization capture reactions were conducted using Dynabeads M-279 (Life Technologies, San Diego, CA) and the xGen Lockdown hybridization and wash kit (Integrated DNA Technologies) according to the manufacturer’s protocols. Captured libraries were amplified on-beads using Illumina p5 and p7 primers in KAPA HiFi HotStart ReadyMix (KAPA Biosystems), followed by purification with Agencourt AMPure XP beads. Library quantification was performed using quantitative real-time PCR with the KAPA Library Quantification kit (KAPA Biosystems), and library fragment size was determined using the Bioanalyzer 2100 (Agilent Technologies, Santa Clara, CA).

### 2.2 Targeted next generation sequencing and data processing

Sequencing was performed on the Illumina HiSeq4000 platform (Illumina, San Diego, CA), followed by data analysis as previously described (10, 11). In brief, low-quality reads (quality <15) and N bases were removed using Trimmomatic. The cleaned reads were mapped to the human reference genome (hg19) using the Burrows-Wheeler Aligner (BWA) (12). PCR duplicates were removed using Picard (https://broadinstitute.github.io/picard/). Local realignment around indels and base quality score recalibration were performed using the Genome Analysis Toolkit (GATK). Single nucleotide polymorphisms (SNPs) and indels were called using VarScan2 and HaplotypeCaller/UnifiedGenotyper in GATK. The mutant allele frequency cutoff was set at 0.5% for tissue samples and 0.1% for cell-free DNA samples, with a minimum of three unique mutant reads required (13). Common SNPs with a population frequency >1% in the 1000 Genomes Project or the Exome Aggregation Consortium (ExAC) 65,000 exomes database were excluded. The resulting mutation list was further filtered using an in-house list of recurrent artifacts based on a normal pool of whole blood samples. Gene fusions were identified using FACTERA (14).

Tumor mutation burden (TMB) was calculated as the number of non-silent somatic mutations per megabase of coding region sequenced. Microsatellite (MS) status was determined using a proprietary in-house developed microsatellite instability (MSI) analysis pipeline. A total of 108 mononucleotide repeats were evaluated, with 52 loci (≥15 bp repeats) identified as MSI determination sites in the targeted sequencing region. These included conventional MSI detection sites such as BAT-25, BAT-26, NR-21, NR-24, and MONO-27. A site was considered qualified for analysis only if it had a coverage depth >100×. A sample was classified as microsatellite instable (MSI) if ≥40% of the qualified MS loci displayed instability, or as microsatellite stable (MSS) if <40% of the qualified MS loci displayed instability, as previously described (15).

## 3 Case presentation

The patient is a 29-year-old Chinese woman. She had no history of urological or gynecological conditions, nor any surgical, sexual, marital, or childbirth history. Her menstrual cycle last about 5 days with moderate flow and no dysmenorrhea. She denied any family history of tumors or related diseases and reported no weight loss. She discovered a pelvic tumor, approximately 6 × 6 cm in size, during a routine physical examination. The tumor did not affect her daily life. As a result, the patient initially opted against aggressive surgical treatment. One year later, the patient underwent another physical examination. While the tumor’s position remained largely unchanged, it had grown rapidly to approximately 6 × 8 cm. Consequently, the patient decided to proceed with surgical removal.

Preoperative laboratory tests showed that the patient’s blood count, liver and kidney function, sex hormones, and markers for infection and tumors were all within normal ranges. Physical examination revealed a firm mass, approximately 6 × 8 cm in size, in the lower abdomen. It was non-tender, with no fluctuation or tenderness. There was no definitive evidence to support a diagnosis of polycystic ovary syndrome (PCOS). In addition, the patient’s anti-Müllerian hormone (AMH) level was elevated at 14.34 ng/mL, above the normal range of 0.89–9.85 ng/mL, which is often seen in PCOS but not conclusive by itself. Enhanced magnetic resonance imaging (MRI) of the lower abdomen revealed a circular mass measuring approximately 76 × 52 × 59 mm. It showed equal signal intensity on both T1 and T2 sequences, a uniform signal on diffusion-weighted imaging (DWI), and a slightly higher signal on apparent diffusion coefficient (ADC) imaging (**Supplementary Figures 1A-D**). Computed tomography (CT) of the pelvis revealed a circular mass, approximately 7.9×5.5×5.3 cm, located in front of the uterus and bladder, just above the posterior symphysis pubis and to the left of the posterior abdominal wall. The mass had clear, smooth borders and a CT value of approximately 41 HU (**Supplementary Figure 1E**).

**Figure 1:**
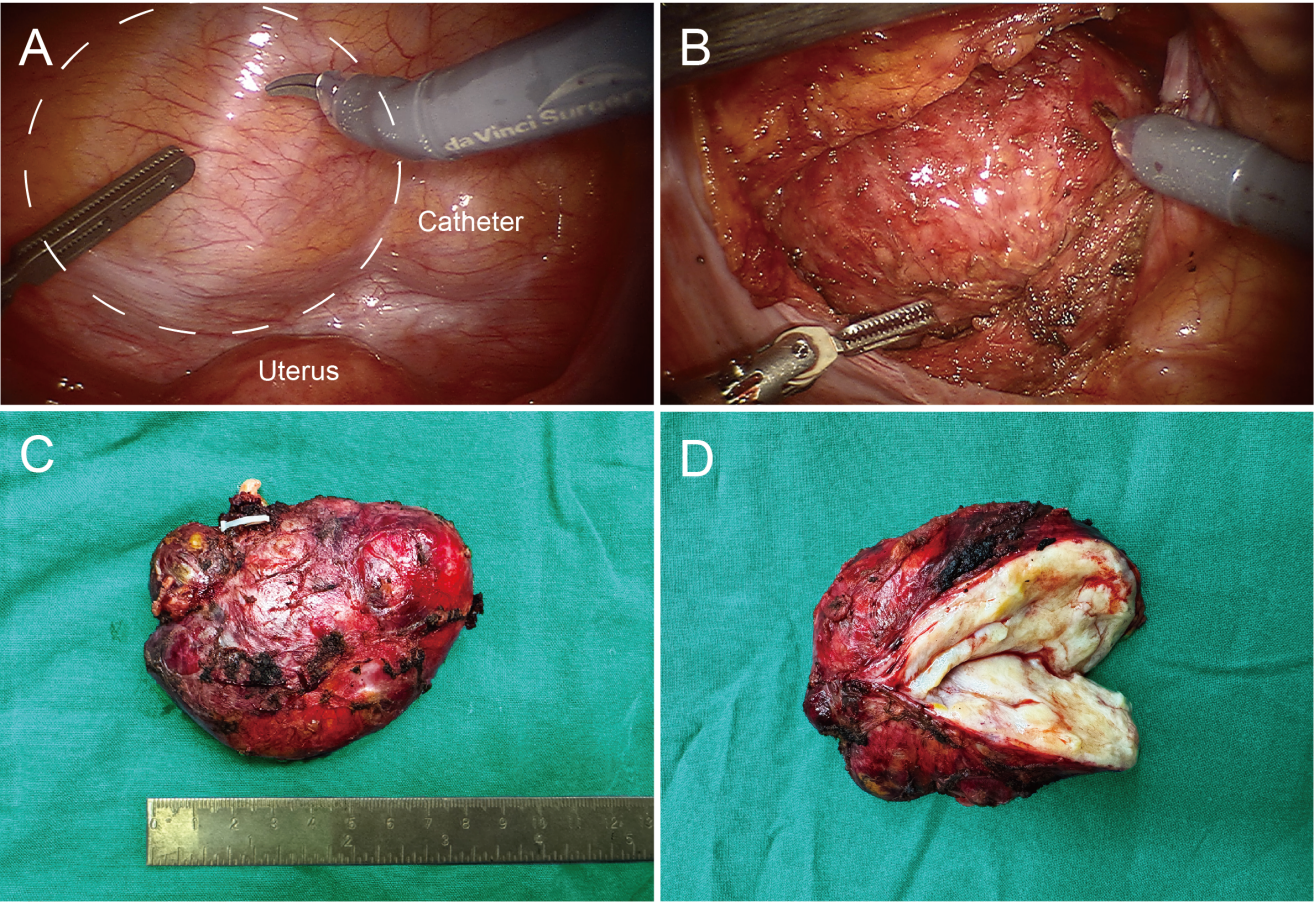
Intraoperative images and postoperative pathological images of pelvic mass. **(A)** Dotted lines outline the tumor’s location, with the uterus and catheter balloon marked for reference. **(B)** The pelvic floor peritoneum was incised, revealing the tumor with a smooth surface and an intact capsule. **(C)** The tumor, measuring approximately 6×8 cm, with an intact capsule. **(D)** Clinical dissection view, showing some firm areas and predominantly soft tissue.

Preoperative imaging revealed an intact tumor capsule without evidence of distant metastasis. Given the risk of iatrogenic tumor seeding, preoperative biopsy was deliberately avoided. Based on the diagnostic findings, the mass was considered likely benign. After consulting with the patient and conducting a thorough preoperative evaluation, the decision was made to proceed with surgical resection assisted by the da Vinci surgical system. The patient was positioned supine, and pneumoperitoneum was established. The pressure was set to 12 mmHg, and the robotic arm was connected. Intraoperative exploration revealed that the tumor was located in the pelvic cavity, extraperitoneal, round in shape, approximately 6×8 cm in size, and appeared to originate from either the bladder or vagina. The pelvic floor peritoneum was incised to access the vesicouterine pouch, and the space between the bladder neck and the anterior wall of the uterus and vagina was carefully separated, while preserving the integrity of the tumor capsule (**Figures 1A, B**). The total operation time was approximately 300 minutes, with setup taking about 30 minutes. Intraoperative blood loss was around 50 ml. The tumor was completely excised without residue, with an intact capsule, smooth surface, and a size of approximately 6x8 cm (**Figures 1C, D**).

The patient started mobilizing on the first day after surgery, began having bowel movements on the second day, and tolerated liquid intake without experiencing severe pain. Given the proximity of the tumor to the bladder, the catheter was retained until the fourth day post-surgery. After removal, the patient resumed normal urinary function. CT examination on the fourth day after surgery showed no residues (**Supplementary Figure 2**). The patient was discharged on the sixth day and followed up for three months without complications or recurrence. The timeline with relevant data from the episode of care is shown in **Table 1**.

**Table 1:** The timeline with relevant data from the episode of care.

LOS Days after the operation	1	2	3	4	5	6	7	8	9
	-	-	-	1	2	3	4	5	6
T (°C)	36.5	36.8	36.6	36.7	36.8	36.7	36.4	36.6	Discharged
P (times)	78	80	90	100	80	71	76	76	
R (times)	16	16	18	18	16	16	16	16	
Blood pressure (mmHg)	91/60	106/62	130/66	125/71	143/68	-	-	-	
Weight (kg)	55	-	-	-	-	-	-	-	
Height (cm)	165	-	-	-	-	-	-	-	
Pain intensity	0	0	0	1	0	0	0	1	
Volume of drainage (mL)	-	-	-	5	150	100	200	90	

LOS, Length of stay; T, temperature; P, pulse; R, respiratory rates.

**Figure 2:**
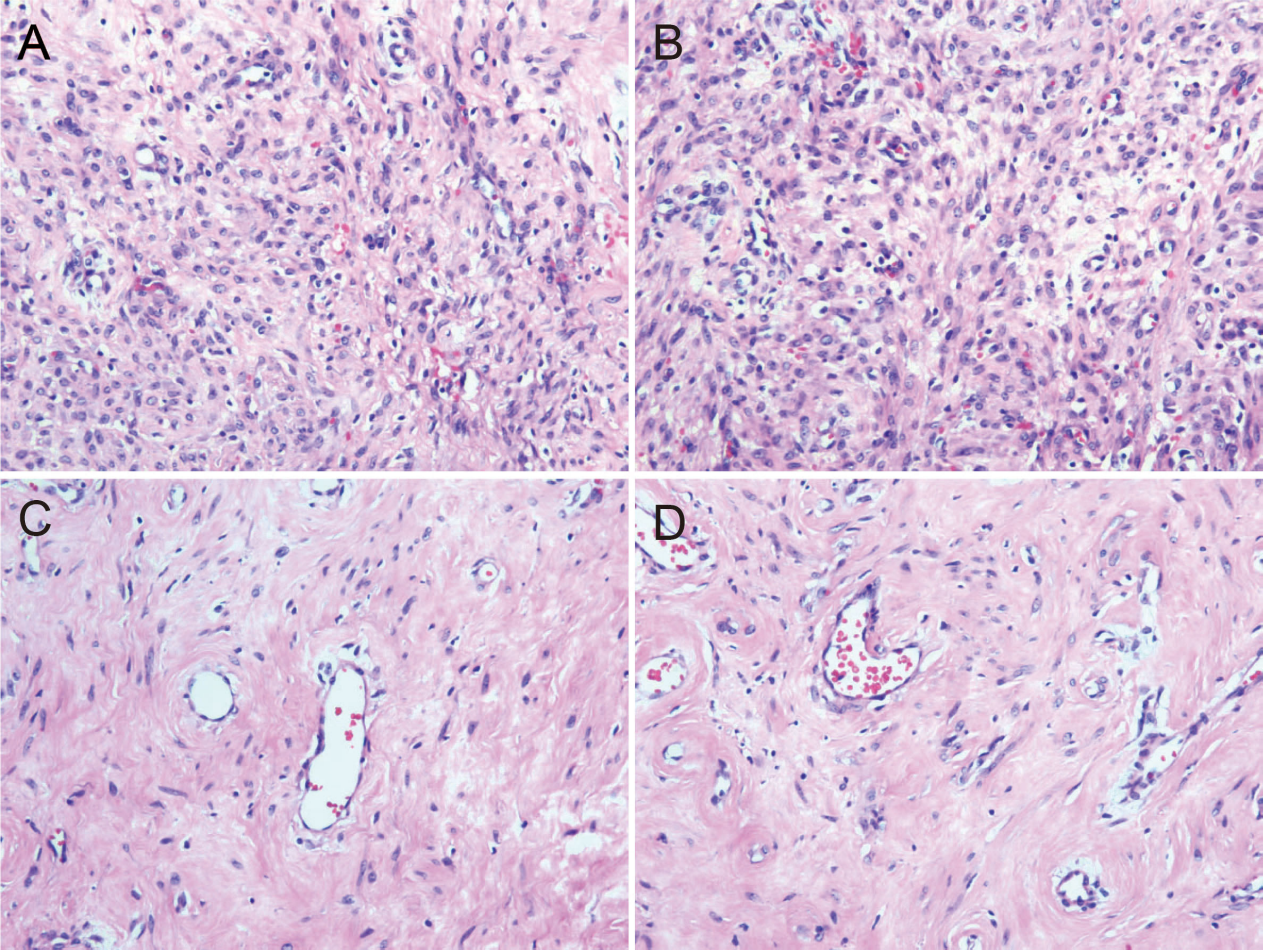
Histopathological pictures (HE 10×20). **(A)** The cell-rich area of angiomyfibroblastoma (AMFB) showed plat spindle-shaped, epithelioid or plasmacytoid cells growing in clusters or small nests around blood vessels. **(B)** The surrounding stroma of angiomyofibroblastoma (AMFB) was loose and edematous. **(C, D)** There were varying degrees of collagen between the tumor cells in the cell-rich area of angiomyofibroblastoma (AMFB). The tumor cells characteristically grew around blood vessels.

**Figure 3:**
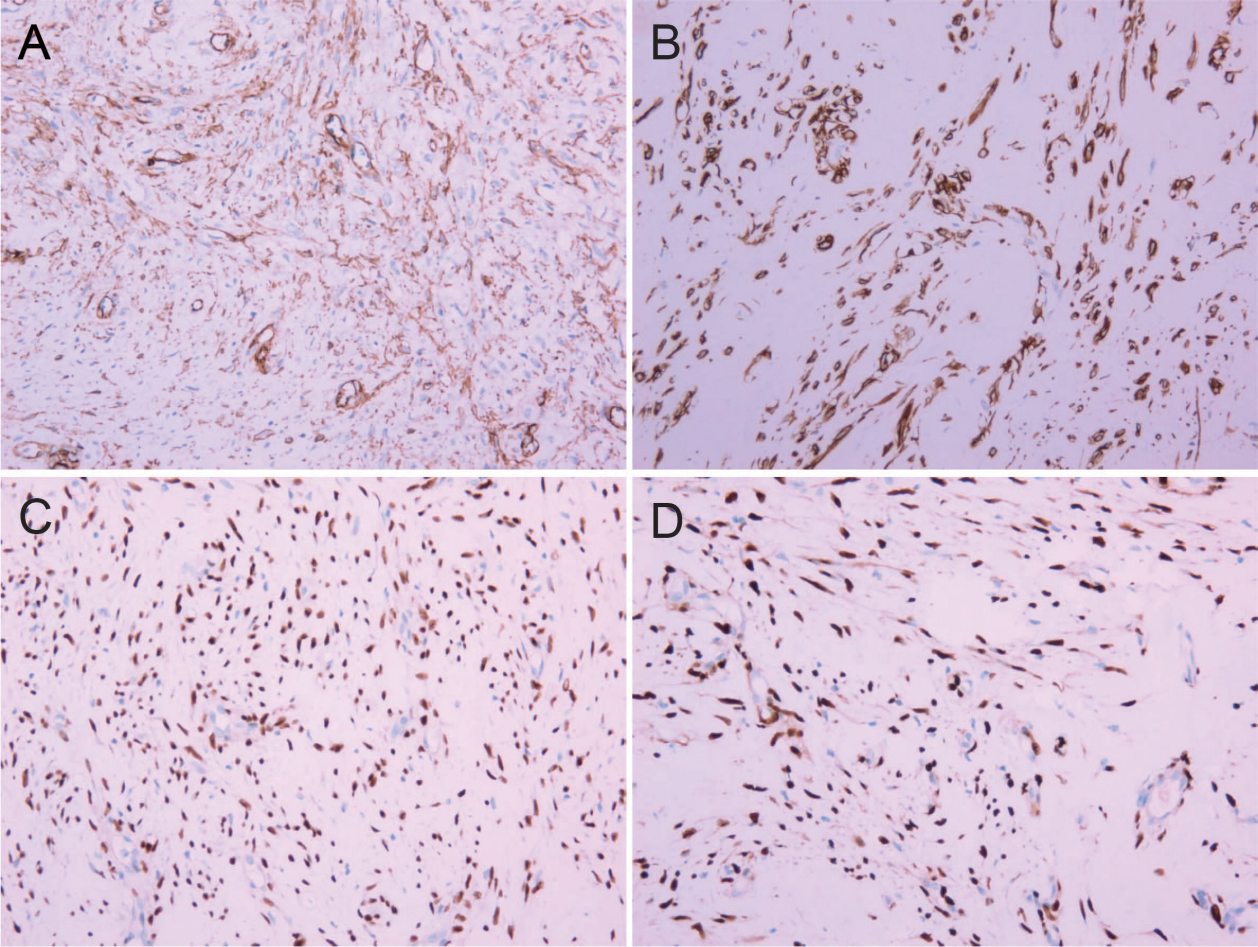
IHC pictures (IHC 10×20). **(A)** CD34(+). **(B)** Desmin(+). **(C)** ER(+). **(D)** PR(+).

**Figure 4:**
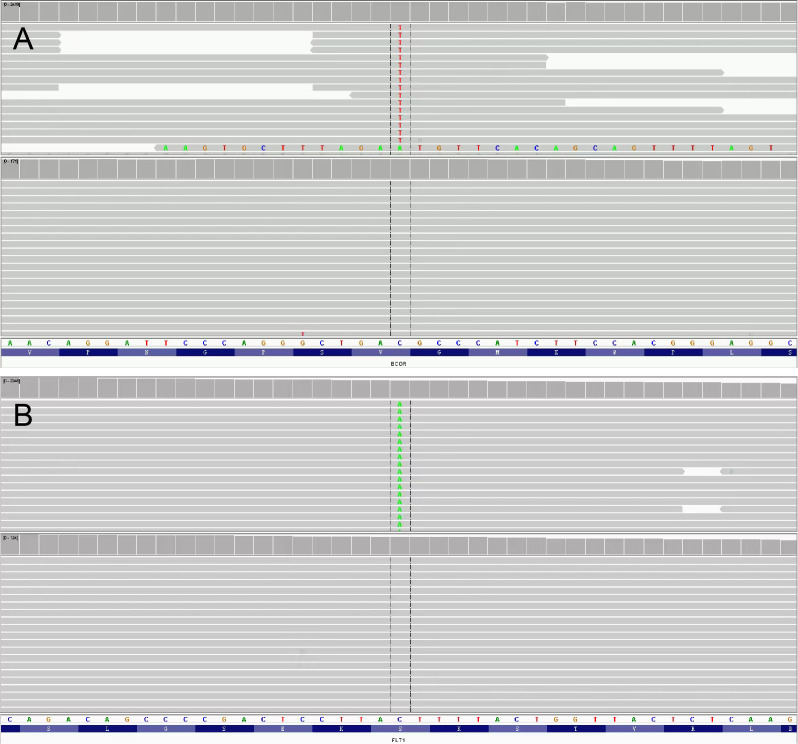
The key mutant spectrum of NGS. **(A)** *BCOR.* **(B)** *FLT1*.

The resected tumor exhibited a gray-white cut surface with rubbery firmness, containing focal areas of soft texture with fasciculated architecture. Although the capsule appeared grossly intact, focal capsular invasion was suspected. The histopathological characteristics are demonstrated in **Figure 2**. Histopathological analysis confirmed the diagnosis. Microscopy revealed a spindle cell tumor, with regions exhibiting histological characteristics similar to cellular angiofibroma. Immunohistochemical analysis revealed the following results: AE1/AE3 (-), STAT6 (-), SMA (-), CD34 (+), Desmin (+), S-100 (weakly positive), CD117 (weakly positive), Bcl-2 (+), DOG1 (-), ER (+), PR (+), HMB45 (-), Caldesmon (-), and Ki-67 (10% positive). **Figure 3** illustrates the expression picture of key IHC molecules. The pathological findings suggested a diagnosis of either AMFB or aggressive angiomyxoma (AA), with HMGA2 serving as a critical marker for differentiation. Since diseases with HMGA2 as a key gene are extremely rare, obtaining and using HMGA2 antibodies is extremely difficult. Therefore, neither the department nor external laboratories were able to perform immunohistochemical analysis for HMGA2. As an alternative, we opted for next-generation sequencing (NGS) to provide a more comprehensive and accurate diagnostic approach. NGS is increasingly used in clinical diagnostics, particularly in oncology and genetics, where it offers the ability to detect a broad range of genetic alterations with high sensitivity and specificity. The sequencing results confirmed the absence of *HMGA2* expression in this case, supporting a diagnosis of AMFB with relatively inactive proliferation. Summary of NGS results are presented in **Table 2**. The key mutant spectrum of NGS is shown in **Figure 4**.

**Table 2:** Summary of NGS results.

Gene	Detection result
*HMGA2*	-
*BCOR* (Rearrangement)	-
*BCOR* (Mutation)	Missense mutation of exon4 of p.V293I
*EWSR1*	-
*FBXW7*	-
*FLT1*	Missense mutation of exon30 of p.S1279I

## 4 Discussion

Primary retroperitoneal tumors have an incidence of 0.5 to 1.0 cases per 100,000 individuals (1). Pelvic tumors, which can arise from internal organs, blood vessels, or the abdominal wall, present significant diagnostic and treatment challenges due to their unique location and proximity to adjacent organs. For preliminary diagnosis and assessment, enhanced CT, MRI, and ultrasound are the preferred imaging methods (2, 3). These imaging techniques help determine whether the tumor is intraperitoneal or extraperitoneal in origin, as well as assess its nature, such as whether it is cystic or solid (3).

The differential diagnosis of pelvic tumors involves a variety of benign conditions, including pelvic infections, gastrointestinal stromal tumors, ectopic pregnancy, endometriosis, leiomyomas, benign neurogenic tumors, and invasive angiomyxomas. Other possible diagnoses include developmental, lymphatic, and interstitial tumors, as well as urogenital tuberculosis and lipomas. Malignant pelvic tumors are typically urogenital and gastrointestinal cancers, angiosarcoma, leiomyosarcoma, synovial sarcoma, malignant neurogenic tumors, and secondary tumors. Additionally, local double-stage malignant mesothelioma has emerged as an increasingly recognized pelvic tumor (3, 4, 16–21).

CT or ultrasound-guided biopsy can aid in confirming the diagnosis of pelvic tumors, though biopsy procedures carry inherent risks, such as potential damage to adjacent organs and the possibility of tumor cell seeding or metastasis (1). Therefore, histopathological examination remains the gold standard for definitive diagnosis.

Angiomyofibroblastoma (AMFB) is a rare, slow-growing interstitial tumor with an indolent clinical course. Since it was first reported in 1992, there have been fewer than 150 cases. Most angiomyofibroblastomas are benign tumors with well-defined boundaries. They primarily occur in the lower reproductive tract of women of reproductive age, although cases have also been reported in the right ventricle and, rarely, in male patients (4–9). In our case, the tumor was located near both the uterus and the anterior bladder, presenting in a rare and complex anatomical position (posterior peritoneum) that required an extensive differential diagnosis to prevent misdiagnosis or overlooked conditions. **Table 3** summarizes the reports of AMFB occurring in non-vulvovaginal sites from 1994 to the present. The histopathological features of these nonvulvar AMFB tumors are summarized in **Supplementary Table 1**. Currently, the specific etiology and mechanisms underlying AMFB remain unclear. However, studies suggest that most cases of AMFB are characterized by “*MTG1-CYP2E1”* fusion genes, and the development of the tumor may be associated with the presence of these fusion molecules (24). Some reports suggest that malignant transformation of AMFB can exhibit high-grade sarcomatoid features, resembling certain sarcomas (25). Histopathological examination of AMFB typically reveals alternating regions of cellular proliferation and atrophy. Immunohistochemical analysis often shows positive staining for Desmin, estrogen receptor (ER), progesterone receptor (PR), and retinoblastoma protein (pRb), while markers such as CD34, Caldesmon, and HMGA2 are typically negative (22, 24). Molecular characteristics can help narrow the differential diagnosis of AMFB, but a comprehensive differential diagnosis still depends on histopathology and immunohistochemistry (7). Cellular angiofibroma and myofibroblastoma share similar histopathological features. Molecular analysis reveals loss of nuclear RB1 expression and deletion of the 13q14 locus by F.I.S.H., necessitating their distinction using desmin immunostaining. AA typically retains nuclear RB1 expression and is HMGA2 positive, whereas myofibroblastoma characteristically overexpresses CD34. Cellular angiofibroma demonstrates loss of nuclear RB1 immunoreactivity. AMFB exhibits significant histopathological differences compared to both AA and cellular angiofibroma. Unlike cellular angiofibroma, AMFB does not demonstrate monoallelic deletions of *RB1* and *FOXO*1 at the 13q14 locus. However, the *MTG1-CYP2E1* fusion transcript is frequently identifiable in AMFB (23). **Table 4** summarizes the differential characteristics of AMFB compared to other soft tissue tumors. The immunoexpression of CD34 and the tumor cells characteristically grew around blood vessels are useful in the distinction from myofibroblastoma to AMFB (22, 23). In this case, the primary differential diagnoses were cellular angiofibroma and invasive angiomyxoma (5). Based on IHC results of CD34(+) and Desmin(+), cellular angiofibroma had been ruled out, therefore, the key point of differentiation in this case was between AMFB and AA. Given the similarities in clinical presentation and imaging findings between the two, histopathology and immunohistochemistry were crucial for an accurate diagnosis (7, 8, 22, 26–28). The immunoreactivity of HMGA2 in a large vulvovaginal mesenchymal lesion confirmed that HMGA2 is a useful biomarker for vulvovaginal invasive hemangiomyxoma, but it may be positive in other mesenchymal lesions in this site (29). As a result, NGS was utilized as an alternative diagnostic tool, confirming the absence of *HMGA2* expression, which was consistent with the diagnosis of AMFB. This technology had better sensitivity than IHC through multigene analysis, which was essential to guide the diagnostic decision process.

**Table 3:** Reports of AMFB occurring in non-vulvovaginal sites: cases published in the literature from 1994 to the present.

Publication year	Age	Gender	Site	Symptoms	Size (cm)	Surgical approach
1994 (30)	40	F	Cervix	-	1.5	-
1997 (31)	39	F	Perineum	-	-	Simple excision
1997 (31)	43	F	The right inguinal region (Nuck tube)	-	4	Simple excision
1999 (32)	24	F	Urethra	Dysuresia	3	Simple excision
1999 (33)	28	F	Fallopian tube	Secondary infertility	25	Simple excision
1999 (34)	46	F	Perineum	Rectovaginal pain	3.5	Simple excision
2007 (35)	28	F	Retroperitoneum	Nausea, vomiting, and pelvic pain	7	Simple excision
2008 (36)	48	F	Retrovesical space	None	3.8	Simple excision
2008 (37)	15	F	Oral cavity	None	3.5	Simple excision
2010 (38)	49	F	Ischiorectal fossa	-	4.2	Simple excision
2011 (39)	44	F	Cervix	-	-	-
2013 (40)	74	M	Nasal cavity	-	2	Simple excision
2014 (41)	-	-	Urethra	-	-	Simple excision
2016 (42)	73	M	Mediastin-um	Dysphagia	9.3	Simple excision
2017 (43)	26	F	Bladder	Hematuria	9.2	Simple excision
2017 (44)	47	F	Broad ligament	Pelvic pain, irregular menstruation	3.5	Laparotomy
2017 (45)	32	F	Cervix	-	1.2	-
2020 (46)	29	F	Pelvis	Pelvic heaviness sensation	15	Laparotomy
2020 (47)	36	F	Fallopian tube	Abdominal pain	6.2	Laparoscopic
2021 (44)	49	F	Broad ligament	-	7.5	-
2022 (22)	25	F	Retroperit-oneum	None	16.9	Partial nephrectomy
2022 (8)	42	M	Scrotum	Scrotal swelling	5	Simple excision
2023 (48)	64	M	Scrotum	Scrotal swelling, frequent micturition	9.4	Simple excision
2024 (49)	51	F	Cervix	None	12	Laparoscopic
2024 (4)	61	M	Between the abdomen and pelvis, bladder and rectum	Dysuria and perineal swelling	20	Robot-assisted resection
2024 (50)	33	M	The inguinal region	None	6.6	Simple excision

F indicates female. M, male. - means not indicated in the literature.

**Table 4:** Differential characteristics of AMFB compared to other soft tissue tumors*.

Tumor type	Histopathologic features	Immunohistochemical reaction		Molecular analyses
		+	-	
Angiomyofibroblastoma	Variably cellular tissue exhibiting abundant thin-walled blood vessels, increased perivascular cellularity, and a collagenous or edematous stroma	Desmin (strong and diffuse, may decrease after menopause), ER, PR, pRb	CD34 (~ 15% positive), SMA (~ 15% positive), HMGA2	*MTG1–CYP2E1* fusion transcripts, *HMGA2(-)*
Cellular angiofibroma	Loosely arranged lipomatoid spindle cells with prominent thick-walled vessels and a fine collagenous stroma	CD34 (30%-60%) ER and PR (50%-60%)	pRb, SMA (~ 25% positive), Desmin (~ 5% positive), HMGA2, S100	Monoallelic loss of *RB1* and *FOXO1* at the 13q14 locus (F.I.S.H.)
Aggressive angiomyxoma	Hypocellular hypervascular neoplasms with mucoid stroma, commonly located in deep genital tissues. Vessels are variable in caliber and typically thick-walled	ER, PR, pRb, CDK4, HMGA2 (86%)	S100	*HMGA2* rearrangements
Myofibroblastoma	Typically well-circumscribed (rarely locally infiltrative) neoplasms with variable cellularity. Low mitotic rate (<1/10 HPFs), small-to-medium vessels with frequent hyalinization, and variably interspersed mast cells	Desmin, CD99, CD34, Bcl-2, ER and PR	α-SMA, S100, pRb	Monoallelic loss of *RB1* and *FOXO1* at the 13q14 locus (F.I.S.H.); *MTG1–CYP2E1* fusion transcripts

*Data from reference (22, 23). CDK4, cyclin-dependent kinase 4; ER, estrogen receptor; PR, progesterone receptor; pRb, retinoblastoma protein; SMA, smooth muscle actin; HMGA2, high mobility group protein A2.

While angiomyofibroblastoma (AMFB) is uniformly benign in biological behavior, as evidenced by the low Ki-67 proliferation index (10%) in our case and excellent post-resection outcomes reported in literature, its histological overlap with more aggressive mesenchymal tumors like aggressive angiomyxoma creates persistent diagnostic challenges, particularly in unusual anatomical locations such as the retroperitoneum. Our case highlights three key clinical implications: first, it demonstrates that even tumors with classic AMFB morphology may require molecular confirmation when presenting in atypical locations; second, it provides a diagnostic framework combining histopathology, immunohistochemistry and next-generation sequencing (NGS) for challenging cases in well-resourced settings; and third, it reaffirms that complete surgical excision with organ preservation remains the therapeutic cornerstone regardless of diagnostic approach. The judicious application of NGS in our diagnostic workflow proved particularly valuable when critical discriminative markers like HMGA2 were technically challenging to evaluate by immunohistochemistry, when the tumor’s retroperitoneal location expanded the differential diagnosis beyond typical vulvovaginal AMFB, and when molecular characterization offered potential therapeutic insights for atypical presentations. However, we emphasize that NGS should complement rather than replace meticulous histopathological evaluation, serving as a valuable adjunct in carefully selected diagnostically ambiguous cases, while its routine use for conventional vulvovaginal AMFB would represent unnecessary resource utilization in most clinical settings.

Surgical resection remains the primary treatment for AMFB, with reports indicating effective outcomes, including virtually no recurrence and metastasis (5, 47). However, specific guidelines for the surgical treatment of tumors at this site have yet to be established. The ideal approach aims to preserve pelvic floor muscles, nerves, and blood vessels while ensuring complete tumor removal and preserving the function of adjacent organs (4). However, the surgery is particularly challenging due to the “funnel effect” of the pelvic cavity, where the narrowing pelvic space adds significant complexity to the procedure. The Da Vinci robotic system, with its high flexibility, precise control, and enhanced visual magnification, can effectively address such challenges, offering unique advantages over both open and laparoscopic surgery (51). Most studies report no significant differences in intraoperative complications, conversion rates, or long-term outcomes between robotic and laparoscopic abdominal and pelvic surgeries. However, the Da Vinci robotic system can be considered the optimal surgical approach for managing the funnel effect of pelvic tumors, excluding considerations of cost and operative time (4).

The reliance on a single case report in this study has inherent limitations and should be acknowledged. To minimize the possibility of misdiagnosis, further investigation of AMFB in a larger cohort is warranted. Additionally, the relatively short follow-up period prevents any conclusions regarding the potential for future relapse or metastasis.

## 5 Conclusion

In summary, we report a rare case of pelvic retroperitoneal angiomyofibroblastoma tumor - AMFB. Due to its rarity in the lower female reproductive tract and its resemblance to other common tumors, it has a broad differential diagnosis. Accurate identification is critical to prevent misdiagnosis. In general, immunohistochemical analysis, when combined with the appropriate morphological features, can provide valuable diagnostic insights. In this case, however, NGS offers an effective alternative diagnostic approach that not only minimizes tissue waste but, more importantly, aids in accurate diagnosis. Complete surgical resection while preserving pelvic floor function is essential. This study contributes to the characterization of AMFB and establishes a foundation for its diagnosis and treatment. However, this paper has some limitations, such as the lack of large sample verification and long-term follow-up data, which needs further research.

## Data Availability

The original contributions presented in the study are included in the article/**Supplementary Material**. Further inquiries can be directed to the corresponding author.
