# Supplementary material for: A case report and literature review: Diagnosis of pelvic retroperitoneal angiomyofibroblastoma assisted by next-generation sequencing
Source: Front Oncol. 2025 Sep 10;15:1560543. doi: 10.3389/fonc.2025.1560543 (PMC12457169; doi:10.3389/fonc.2025.1560543)
Supplement: Supplementary file 1 [file Image1.pdf]

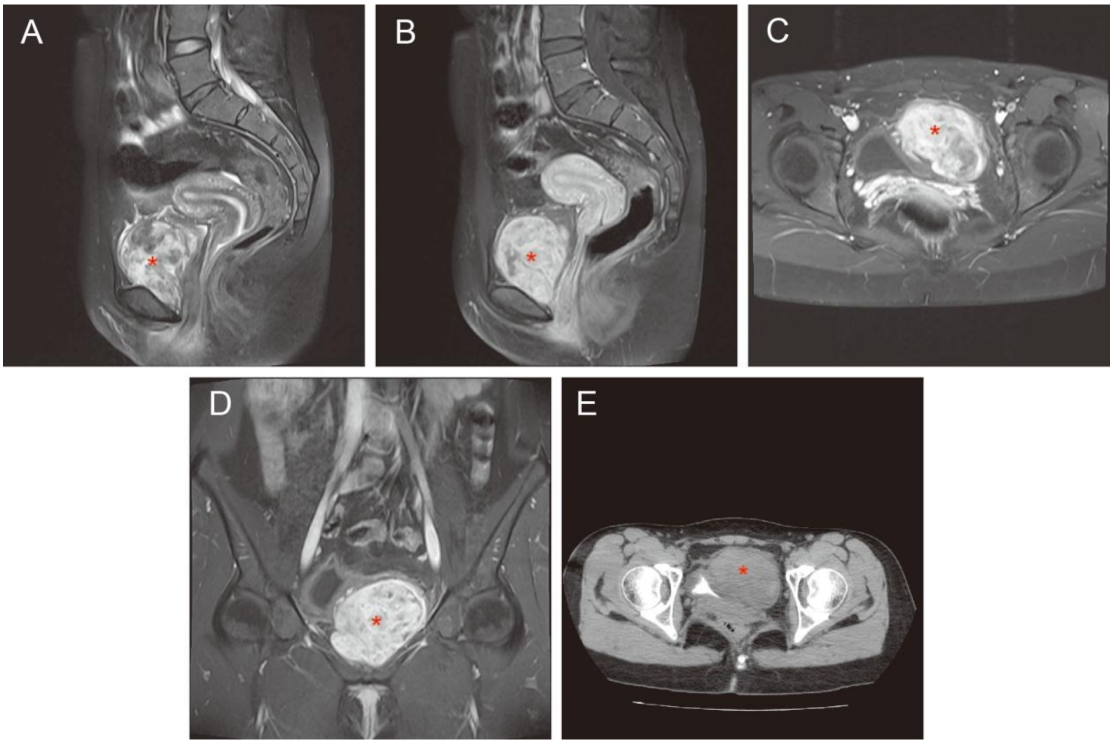

Supplementary Figure 1. Panels A-D show preoperative enhanced MRI images of the patient. A round mass, approximately 76×52×59 mm in size, was identified in the left anterior and lower portion of the uterus. The mass exhibited equal signal intensity on T1 and T2, a uniform signal on DWI, and a slightly higher signal on apparent diffusion coefficient (ADC). It had clear boundaries with significant uniform enhancement and a distinct demarcation from the uterine wall. The lower margin extended to the symphysis pubis. Imaging diagnosis: Solid mass in the left anterior and higher part of the uterus. Myoma with hyaline degeneration and edema was considered. E. Preoperative CT images of the patient. A circular mass, measuring approximately 7.9×5.5×5.3 cm, is located in front of the uterus and an unfilled bladder, above the posterior symphysis pubis, and to the left of the abdominal wall. The mass has clear, smooth boundaries, with a CT value of approximately 41 HU. Imaging diagnosis: Pelvic mass, likely benign. *Indicates the tumor location.
