# Supplementary material for: A case report and literature review: Diagnosis of pelvic retroperitoneal angiomyofibroblastoma assisted by next-generation sequencing
Source: Front Oncol. 2025 Sep 10;15:1560543. doi: 10.3389/fonc.2025.1560543 (PMC12457169; doi:10.3389/fonc.2025.1560543)
Supplement: Supplementary file 2 [file Image2.pdf]

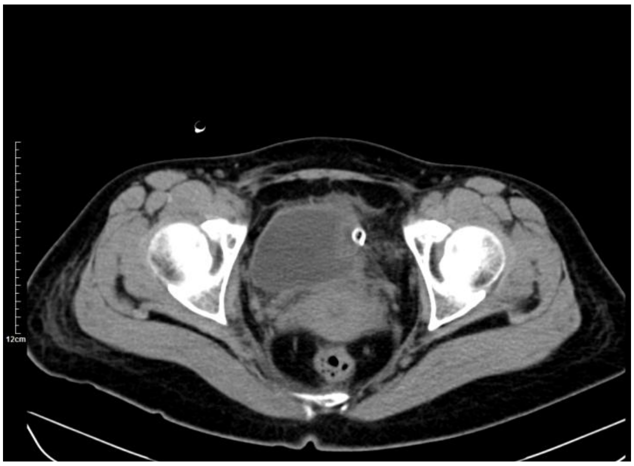

Supplementary Figure 2. Postoperative imaging examination. Abdominal CT examination on the fourth day after surgery. The bladder was well distended with a smooth, uniform wall, and no obvious abnormalities were observed inside. The images revealed no tumor residue, with the circular high-density area identified as a rubber drainage tube.
