# Supplementary material for: A case report and literature review: Diagnosis of pelvic retroperitoneal angiomyofibroblastoma assisted by next-generation sequencing
Source: Front Oncol. 2025 Sep 10;15:1560543. doi: 10.3389/fonc.2025.1560543 (PMC12457169; doi:10.3389/fonc.2025.1560543)
Supplement: Supplementary file 3 [file Table1.pdf]

Supplementary Table 1. Histopathological report of AMFB occurring in non-vulvovaginal sites: cases published in the literature from 1994 to the present.

Publication year	Site	Gross features	Histopathologic	Immunohistochemistry
1994[29]	Cervix	-	-	-
1997[30]	Perineum	-	-	-
1997[30]	The right inguinal region (Nuck tube)	-	-	-
1999[31]	Urethra	The tumor was soft in texture, with a smooth and shiny yellowish brown surface, and there were no areas of necrosis in transverse section.	The lesions showed vascular and perivascular proliferation of spindle cells with oval or round nuclei and fine chromatin. Stromal cells were interspersed between wavy collagen fibers and capillaries. Very few mitotic figures were present.	Desmin (+), SMA (+).
1999[32]	Fallopian tube	The tumor showed alternating zones of cell proliferation and abundant thin-walled blood vessels and spindle cells surrounded by ovoid stromal cells.	The mitotic activity was extremely low.	Vimentin (+), Desmin (+).
1999[33]	Perineum	The tumor was clearly outlined by a thin pseudocapsule.	The mass consisted of alternating areas of hypocellularity and hypercellularity. The cells had an ovoid or spindle-shaped morphology and were embedded in edematous collagenous tissue. The vessels in the tumor were numerous, small and medium sized, and	-

			the wall was thin. Variable numbers of inflammatory cells and extravasated red blood cells were detected throughout the lesion. A large amount of mature adipose tissue was scattered between the tumor areas.	
2007[34]	Retroperitoneum	-	-	-
2008[35]	Retrovesical space	-	Compactly arranged epithelioid ovoid or blunt spindle tumor cells with monotonous small nuclei and eosinophilic cytoplasm. There were prominent dilated vessels, which were surrounded by an eosinophilic or myxoid fibrous matrix.	Actin (+)
2008[36]	Oral cavity	Polypoid lesions with smooth borders and glint on the incisional surface were observed.	Small cell proliferation was seen in the abundant edematous matrix. Many thick hyalinized collagen fibers were scattered throughout the tumor. Alternating hypercellular and hypocellular areas with myxoid/edematous stroma rich in thin-walled vessels. A band of fibrous tissue separated the tumor cells from the overlying squamous epithelial cells. Tumor components were represented by small to medium sized, round to oval to epithelioid cells. A few cells were binucleated or multinucleated epithelioid cells or elongated in shape. Thick hyalinized collagen fibers were scattered throughout the	Vimentin (+), Desmin (+), only focally positive to α-SMA and HHF35. No staining was obtained with Myogenin (Myf-4), Myo-D1, CD34, h-caldesmon, CD99, Cytokeratins, EMA, S-100, HMB45, Bcl-2, and ER/PR receptors.

			tumor.	
2010[37]	Ischiorectal fossa	-	-	-
2011[38]	Cervix	-	-	-
2013[39]	Nasal cavity	-	-	-
2014[40]	Urethra	-	-	-
2016[41]	Mediastin-um	-	The tumor consisted of small cells embedded in a prominent mucoid/edematous matrix, with areas of cytopenia and hypercellularity. Abundant blood vessels of different wall thicknesses were distributed in the collagen matrix. The stroma was composed of spindle-shaped stromal cells with eosinophilic qualities.	Vimentin (+), Desmin (+) and CD34 (+), S-100 (-). The proliferation index (expressed as the percentage of Ki-67 antigen-positive nuclei) was less than 20％.
2017[42]	Bladder	Gross features, it was a well-defined mass. The cut surface was pure off-white and showed the presence of multiple cystic Spaces ranging from 0.2 cm to 1.5 cm in diameter.	The lesion showed irregular vascular proliferation with edematous areas of hypercellularity and hypocellularity. Spindle cells proliferated with oval to round nuclei and fine chromatin. Multiple cystic Spaces lined by flat plain epithelial cells were also observed. A few scattered inflammatory cells, slender collagen bundles, and a myxoid fibrous matrix were also detected in the lesion.	ER (+), Vimentin (+), Bcl-2 (+), Desmin(focally positive). CD34 (-), Pan CK (-), SMA (-), Alk1 (-), HMWCK (-). Mitotic activity was minimal and Ki-67 showed 2% to 3% positivity.
2017[43]	Broad ligament	-	The tumor was composed of ovoid or spindle-shaped tumor cells arranged in bundles or waves around small blood vessels	Vimentin (+), Desmin (+), ER (+), PR (+), S-100 (-), SMA (-).

			in the stroma, with areas of high and low cell density.	
2017[44]	Cervix	-	-	-
2020[45]	Pelvis	Well-defined tumor with no true capsule. The incision surface was yellow-white and uniform without hemorrhage or necrosis.	Histopathological examination appeared to be well demarcated, showing alternating areas of hypercellularity and hypocellularity with abundant blood vessels. There was edema in the stroma. The tumor cells were spindle-shaped with fine chromatin. No mitotic pattern was observed.	ER (+), PR (+), CD34 (+), Desmin (+). SMA (-), PS-100 (-).
2020[46]	Fallopian tube	-	Many vessels were surrounded by spindle and epithelioid cell cords, as well as a loose fibromyxoid matrix, without mitotic atypia.	Vimentin (+), Desmin (+), SMA (+), ER (+), PR (+), CD34 (-), pan-cytokeratin (-).
2021[43]	Broad ligament	-	-	-
2022[22]	Retroperit-oneum	The tumor was well circumscribed without renal parenchyma invasion, and the incision surface was tan, ringed, edematous, and focally calcified.	A localized mass consisting of anastomosed cell islands and bands in a fibrous matrix containing many thin-walled vessels. Tumor cells showed perivascular aggravation, with focal intra-focal fat interspersed in a collagen background. They are plump, epithelial to stellate, and have fine chromatin. No mitotic figures were found.	Desmin (+), ER (+), PR (+), CDK4 (diffuse, cytoplasmic +), S-100 protein (scattered, single-cell +), pRb (+). SMA (-), h-Caldesmon (-), CD34 (-), ALK1 (-), MDM2 (-), STAT6 (-), nuclear β-catenin (-), CD117 (-), Melan-A (-), cathepsin K (-), HMB-45 (-). Ki-67 did not show significant proliferative activity (<1%). Fluorescence in situ hybridization was negative for MDM2 amplification.
2022[8]	Scrotum	The tumors were well encapsulated.	The tumors were rich in thin-walled and small-sized vessels. Hyperplastic spindle cells can be seen around the vessels.	SMA (+), S-100 (-). Desmin (+), a proliferation index of CD34 and Ki67 of approximately 1%.

2023[47]	Scrotum	-	The lesion consisted of uniformly distributed areas of decreased and increased cells. Hyaline vessels of small to medium capillary size were observed in the stroma. In areas of hypercellularity, plain spindle-shaped cells with unclear cytoplasm, short blunt to wavy nuclei, and thin collagen were observed in the stroma. Areas of reduced cells showed edematous stroma with similar blunt spindle cells.	CD34 (+), S-100 (-), SMA (-), Desmin (-). In areas with the highest proliferation, the Mib-1 labeling index ranged from 2 to 4%. Mast cells were distributed among the tumor cells and were highlighted by C-Kit (CD117) immunohistochemistry.
2024[48]	Cervix	The outer surface of the cystic mass was white and glittering. The surface of the incision was white fleshy, with multiple septa and polycystic and intratumoral hemorrhage.	Representative sections revealed a relatively well-defined polycystic tumor with alternating cytopenic and cytopenic lesions. In cytopenic lesions, the stroma is hyalinized and has an edematous appearance. In contrast, in hypercellularity lesions, spindle or oval stromal cells are oriented and clustered around thin-walled vessels. These tumor cells had insipid looking chromatin and inconspicuous nucleoli. There is no mitosis. Occasionally, thick-walled congested vessels were mixed with tumor cells.	The capillary-sized vessels within the tumor were positive for the vessel maker CD31. However, tumor cells were negative for CD31. This suggests that the tumor cells are neither endothelial nor vascular in origin. ER (+), PR (+), SMA (+). CD34 (-), D2-40 (-), Desmin (focally positive).
2024[4]	Between the abdomen and pelvis, bladder and rectum	-	-	-

2024[49]	The inguinal region	-	-	Vimentin (+), SOX10 (-), S-100 (-), MSA (-), Desmin (+), CD34 (+), MyoD1(-), Caldesmon (-), SMA (-), and Ki-67 (+, <1%)

- means not indicated in the literature. α-SMA, α-smooth muscle actin. HHF35, muscle-specific actin. ER, estrogen receptors. PR, progesterone receptors. CD34, cluster of differentiation 34. pRb, retinoblastoma protein.
